# Proinflammatory Soluble Interleukin-15 Receptor Alpha Is Increased in Rheumatoid Arthritis

**DOI:** 10.1155/2012/943156

**Published:** 2012-07-25

**Authors:** Ana Cecilia Machado Diaz, Araceli Chico Capote, Celia Aurora Arrieta Aguero, Yunier Rodríguez Alvarez, Diana García del Barco Herrera, Miguel Estévez del Toro, Gerardo E. Guillen Nieto, Alicia Santos Savio

**Affiliations:** ^1^Pharmaceutical Division, Center for Genetic Engineering and Biotechnology, 10600 Havana, Cuba; ^2^Rheumatology Department, H. Ameijeiras Hospital, San Lazaro 701, 10300 Havana, Cuba

## Abstract

Rheumatoid arthritis (RA) is an autoimmune and inflammatory disease in which many cytokines have been implicated. In particular, IL-15 is a cytokine involved in the inflammatory processes and bone loss. The aim of this study was to investigate the existence in synovial fluid of soluble IL-15R**α**, a private receptor subunit for IL-15 which may act as an enhancer of IL-15-induced proinflammatory cytokines. Soluble IL-15R**α** was quantified by a newly developed enzyme-linked immunosorbent assay (ELISA) in samples of synovial fluid from patients with RA and osteoarthritis (OA). The levels of IL-15R**α** were significantly increased in RA patients compared to OA patients. Also, we studied the presence of membrane-bound IL-15 in cells from synovial fluids, another element necessary to induce pro-inflammatory cytokines through reverse signaling. Interestingly, we found high levels of IL-6 related to high levels of IL-15R**α** in RA but not in OA. Thus, our results evidenced presence of IL-15R**α** in synovial fluids and suggested that its pro-inflammatory effect could be related to induction of IL-6.

## 1. Introduction

Rheumatoid arthritis (RA) is a chronic autoimmune disease in which imbalances in pro- and anti-inflammatory cytokines promote induction of autoimmunity, inflammation and joint destruction [1]. IL-15 is a proinflammatory cytokine associated with several autoimmune diseases, particularly rheumatoid arthritis. [2, 3]. Three different functional forms of IL-15 have been identified: the soluble cytokine [4], IL-15R-independent membrane-bound IL-15 [5, 6] and membrane-IL-15 anchored through IL-15R*α* [7]. IL-15R*α* is a unique high affinity private *α* chain that together with the IL-2 receptor, IL-2R*β* chain and the IL-2R*γ* chain subunits constitute a trimeric receptor for IL-15 on cell membranes. Also, IL-15R*α* may be secreted as a functional soluble molecule (s-IL-15R*α*) and could behave as an agonist by forming a complex with IL-15 which is 100-fold more efficient than the noncomplexed soluble cytokine or as an antagonist for IL-15 [8–10].

In addition, s-IL-15R*α* may activate a reverse signaling through interaction with IL-15R-independent membrane-bound IL-15, activating MAPKs (mitogen-activated protein kinases) and increasing production of several proinflammatory cytokines such as IL-6, IL-8, and tumor necrosis factor *α* [5, 6, 11]. This bidirectional signaling has also been described for most members of TNF ligand family contributing to multiple stages of immune regulation [12].

Soluble IL-15 has been detected in synovia of patients with RA mainly expressed by macrophages, fibroblasts, and endothelial cells [13, 14], and there it recruits circulating memory T cells in the synovial membrane and may up regulate TNF*α*, IL-17, and other proinflammatory cytokines [15–17]. Moreover, soluble IL-15 appears to be an important contributor to osteoclastogenesis contributing to bone erosion [18–20]. Membrane IL-15 has been detected in synovial tissue of RA patients. However, the role of IL-15R-independent membrane-bound IL-15 in RA has not been completely studied until now, and no data has been reported so far concerning the existence of natural sIL-15R*α* in synovial fluid of RA patients, two elements necessary to induce proinflammatory cytokines through reverse signaling that could contribute to pathogenesis of RA. Existence of different IL-15-activating signaling pathways for inducing inflammation in RA could imply use of different antagonists depending on the specific induced pathway(s).

We had previously identified P8 peptide as an IL-15R*α* antagonist that may be inhibiting reverse signaling [21]. Therefore, we focused our study on determining the presence of soluble IL-15R*α* in synovial fluid and its potential role in inducing reverse signaling through membrane-bound IL-15 on cells from synovial fluid. Interestingly, we found higher levels of IL-15R*α* in RA compared with OA, and also we found that there is a positive relationship between these high levels of IL-15R*α* and high levels of IL-6 in RA but not in OA. Furthermore, we demonstrated in an *in vitro* experiment that IL-15R*α* induced secretion of IL-6 in cells from synovial fluid of an RA patient. These results suggest the role of sIL-15R*α* as an inducer of the pro-inflammatory cytokine IL-6 through a reverse signaling in RA.

## 2. Subjects and Methods

### 2.1. Patients and Samples

Synovial fluids were obtained from the knee joints of 35 patients. Eighteen (18) of them with established RA were receiving treatment with oral methotrexate (MTX) and low-dose prednisone. They were moderate or nonresponders to MTX with a mean DAS28 of 4.7 and had shown inflammation and abundant synovial fluid in the cavities of synovial joints. The rest (17) were OA patients. All patients were from the Rheumatology Service at Ameijeiras Brothers Hospital. Permission was obtained from the local ethics committee, and all patients gave written informed consent. Patient demographics are listed in Table 1.

Synovial fluid was directly aspirated from the inflamed joint and collected into tubes, immediately after we added hyaluronidase type IV (H3884, Sigma, USA) at 10 ug/mL to synovial fluid, and mixed by inversion followed by spinning at 1000 g for 10 min within 30 min of sample collection. The acellular portion of synovial fluid (synovial liquid) was stored at −70°C before subsequent analysis. Cells were collected for flow cytometry and cell stimulation experiments.

### 2.2. Measurement of Serum IL-15R*α*

We have developed an enzyme-linked immunosorbent assay (ELISA) format to measure serum levels of IL-15R*α* as we have previously described [21]. The 96-well microtiter plates (Costar, Corning Inc., NY, USA) were treated with 2% glutaraldehyde solution for 2 h at 37°C. After two washes with water, plates were coated with 10 *μ*g/mL of P8 peptide/well, and the plates were then incubated at 4°C overnight. After three washes with phosphate buffered saline pH 7.4 (PBS) containing 0.05% Tween 20, nonspecific binding sites were blocked by incubation for 1 h at 37°C in PBS containing 1% BSA. The blocking solution was replaced by samples (synovial liquid diluted 2-fold in PBS, containing 0.01% BSA and 0.05% Tween 20 or different concentration of recombinant IL-15R*α*-Fc (147-IR, R&D) in the same buffer). All the samples were in triplicate. Following incubation at 37°C for 2 h, we did three washes with PBS containing 0.05% Tween 20. IL-15R*α* was detected with specific antibody against IL-15R*α* (AF247, R&D System). The bound IL-15R*α* was detected with HRP-conjugated goat antihuman IgG (A0170, Sigma, USA) by incubation at 37°C for 1 h, followed by 5 washes with PBS, 0.1% Tween 20. The reaction was visualized by adding the substrate solution (3,3′,5,5′-tetramethylbenzidine [TMB]), and absorbance at 450 nm was measured with an ELISA plate reader (Biotrak GE, Healthcare, USA). The detection limit was 0.25 nM.

### 2.3. Immunoassays for IL-6

Interleukin-6 concentrations were measured in duplicate using commercially available ELISA kits purchased from R&D Systems (Quantikine Human IL-6, D6050). The detection limit was 3.12 pg/mL.

### 2.4. Western Blot Analysis

 We precipitated IL-15R*α* from the synovial fluid with cold acetone. Proteins were separated on 12.5% SDS-PAGE and transferred to nitrocellulose membranes. Membranes were blocked in 5% nonfat dry milk in Tris-buffered saline (TBS) (pH 8) for 1 hour at room temperature before probing for 2 h with antibody against IL-15R*α* (0.5 *μ*g/mL) (AF247, R&D System). After incubation with horseradish-peroxidase- (HRP-) conjugated secondary antibodies (rabbit anti-goat HRP, 1 : 1,000 dilution) (A8919, Sigma, USA) in 5% in TBS (pH 8) for 1 h at room temperature, bound antibodies were visualized using enhanced chemiluminescence (Amersham Pharmacia Biotech, Little Chalfont, UK).

### 2.5. Flow Cytometry

Freshly isolated cells collected by centrifugation from synovial fluid were washed with PBS, 2% fetal calf serum (FCS), and incubated on ice for 1 h with an anti-IL-15 mAb (MAB2471; R&D System), or an irrelevant IgG1 isotype control mAb (MAB002; R&D System), anti-CD3 (sc1239; Santa Cruz Biotechnology), or anti-CD8 (sc 7970; Santa Cruz Biotechnology). Cells were then washed and incubated on ice for 30 minutes with anti-mouse-FITC (F2772, Sigma, USA). After washing once with PBS 2% FCS and once with PBS, cells were resuspended in 1% paraformaldehyde and analyzed in a PAS-III flow cytometer using FloMax software (Partec, Germany).

For acid treatment, cells were incubated in ice-cold glycine buffer (25 mM glycine and 150 mM NaCl [pH 3.0]) for 10 min previously incubated with specific antibodies.

### 2.6. Cell Stimulation with IL-15R*α*

Cells were incubated in 24-well plates at 10^6^ cells per well either with or without sIL-15R*α* at 250 ng/mL as duplicates in two independent experiments. After 72-hour incubation, supernatants were collected and stored at −70°C until further evaluation. IL-6 concentration was determined by ELISA (D6050, R&D Systems, Minneapolis, MN, USA).

### 2.7. Statistics

The nonparametric Mann-Whitney *U* test was used for group comparisons of IL-6 and IL-15R alpha serum levels. The correlation coefficient was obtained by the nonparametric Spearman's rank correlation test.

## 3. Results

### 3.1. Increased SIL-15R*α* Levels in Synovial Fluid from Patients with RA

**Figure 1:**
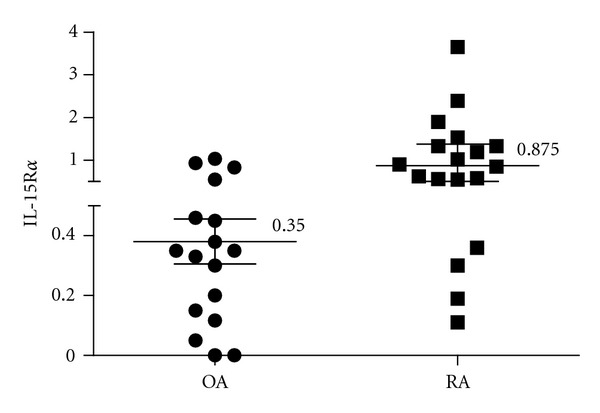
Increased sIL-15R*α* levels in RA synovial fluids. The graphic represents median and interquartile range. Mann-Whitney test shows a significant difference, *P* = 0.0025, between OA and RA groups.

An indirect ELISA assay was performed to measure IL-15R*α* concentration using P8 peptide as capture. Therefore, we detected IL-15R*α* using an anti-IL-15R*α* antibody (AF247, R&D) as a detection antibody as previously described [21]. Human sIL-15R*α*-Fc fusion protein (R&D) was used as standard with a detection limit of 0.25 nM. Next, we measured the IL-15R*α* level in synovial fluid from patients with RA or OA. The sIL-15R*α* was detected in 18 of 18 RA patients (100%) versus 14 of 17 patients of OA (82.3%). A significant increase in concentrations of sIL-15R*α* was observed in synovial fluid collected from RA patients compared to those from OA patients (Figure 1).

**Figure 2:**
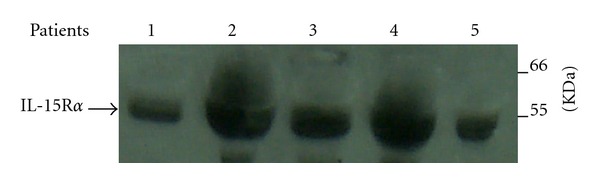
Western blot analysis of IL-15R*α* in synovial fluids from RA patients. Proteins from synovial fluids from different patients (lanes 1–5) were separated on 12.5% SDS-PAGE and transfer to nitrocellulose for western blotting. The western blot was probed with anti-IL-15R*α* antibody and development with anti-goat-HRP. Representative western blot is shown.

To confirm IL-15R*α* protein in synovial fluids, we used P8 peptide synthesized on TentaGel-S pearls to capture IL-15R*α*. Proteins bound to TentaGel-P8 peptide were eluted and analyzed by immunoblotting assay using a specific anti-IL-15R*α* antibody (AF247, R&D). A band was detected between 29 and 66 kDa (Figure 2), corresponding to previously described size around 55 kDa [9].

### 3.2. Synovial Cells Express Membrane IL-15

**Figure 3:**
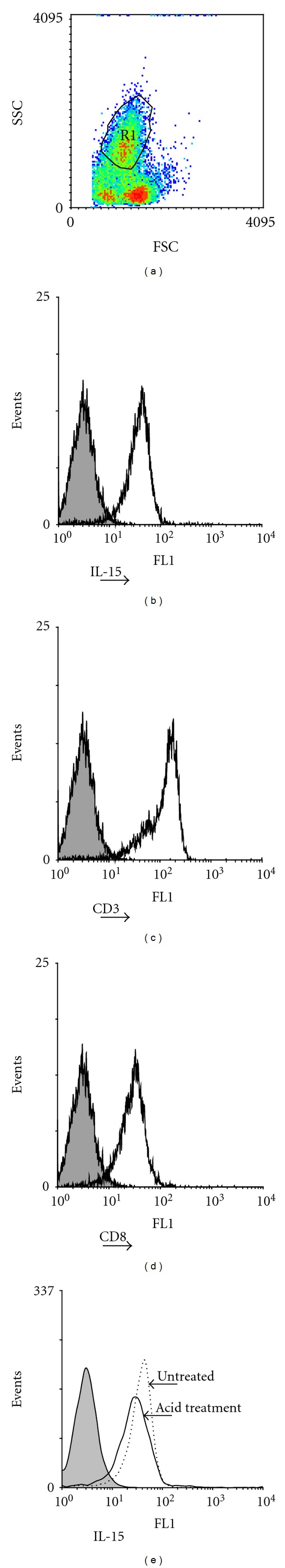
Flow cytometric analysis of a cell population positive for membrane-bound IL-15 (R1 region). Density plot had shown different population of cells in synovial fluid (a). Fluorescence intensity in R1 region is represented by white histograms, using a specific antibody MAB 2471(b); specific antibodies to detect CD3 (c) or CD8 (d) and gray histograms refer to the background staining. Acid treatment with acid buffer (pH 3.0) before incubation with MAB 2471 produced a slight decrease in fluorescence intensity (bold gray line) in comparison to incubation with MAB 2471 in PBS (dotted line) (e).

To study the presence of membrane-bound IL-15, we analyzed the expression of membrane IL-15 by FACS in cells present in the synovial fluid. As shown in Figure 3(a), there is a different cell population in synovial fluids, but we only detected the expression of membrane IL-15 in R1 region (Figure 3(b)). The mIL-15 detected varied among patients, and interestingly, we found that the cell population in R1 region also expresses CD3 (Figure 3(c)) and CD8 markers (Figure 3(d)).

In addition, we tested whether IL-15 was expressed as membrane-IL-15 anchored through IL-15R*α* or as IL-15R-independent membrane-bound IL-15. To accomplish this, we performed acidic treatment to remove membrane-IL-15 anchored through IL-15R*α* as reported by Dubois et al. [7]. This result showed a slight decrease in the amount of cell-surface-bound IL15 after acidic treatment which suggests that part of the protein could be associated to IL-15R*α* in the membrane, but most of IL-15 is expressed as a membrane-anchored protein (Figure 3(e)).

### 3.3. Relationship between IL-6 and IL-15R*α* in Rheumatoid Arthritis

**Figure 4:**
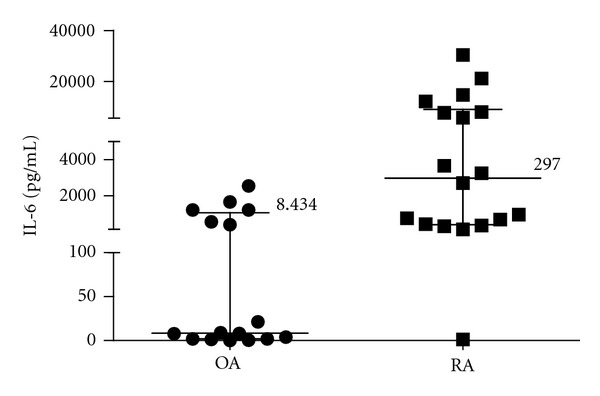
Increased IL-6 levels in RA synovial fluids. The graphic represents median and interquartile range. Mann-Whitney test has shown significant difference *P* = 0.0011, between OA and RA groups.

**Figure 5:**
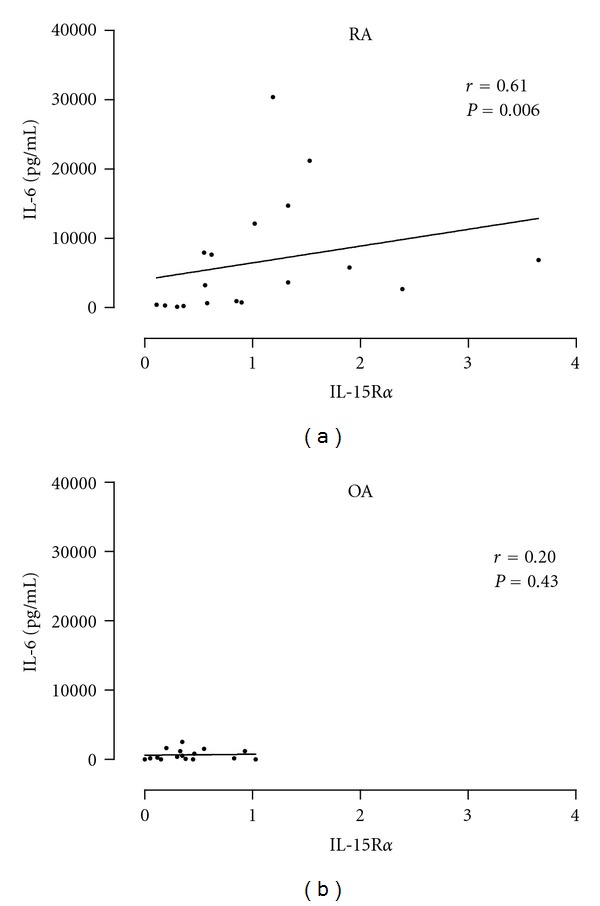
Correlation between IL-6 and sIL-15Ra levels in synovial fluid. Positive correlation was observed in RA (*r* = 0.61).

 IL-6 levels were measured by ELISA in synovial fluids in both groups of patients, and we found that a high percent of RA patients (80%) expressed high levels of IL-6 (>700 pg/mL) versus 35% of OA patients as shown in Figure 4. This result in our patients is in agreement with previous reports but interestingly, in RA but not in OA, synovial IL-6 levels were positively correlated with high levels of sIL-15Ra (*P* = 0.006). The result is showed in Figure 5.

**Figure 6:**
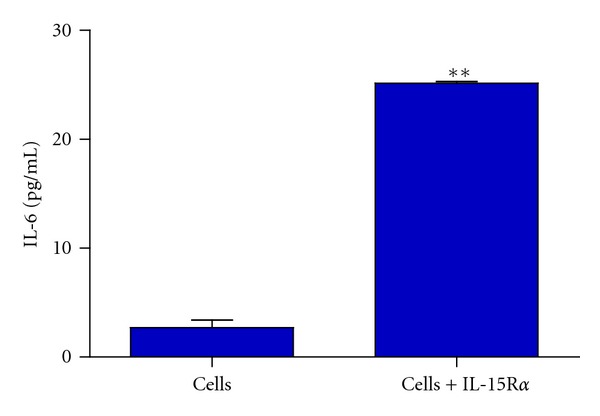
Effect of IL-15R*α* on IL-6 secretion in synovial cells. Treatment with 250 ng/mL of IL-15R*α* significantly upregulated IL-6 expression. Each bar represents the mean and SD of two determinations. ** = *P* < 0.001.

In order to study the induction of IL-6 by IL-15R*α* in cells from synovial fluid, we performed an experiment to incubate cells from synovial fluid of RA patient with IL-15R*α* and in Figure 6 show a strong activation of IL-6 secretion determined by ELISA after 72 h of treatment.

## 4. Discussion

IL-15R*α* is a private receptor for IL-15 that plays an important role in the biology of this cytokine. It has been described as a membrane and soluble receptor in serum from mice and humans [9]. This recent study confirms the presence of soluble IL-15R*α* in synovial fluids from RA and OA patients, although it was undetected in 3 patients of OA. First, we established an ELISA using as a capture a previously described peptide which specifically binds to IL-15R*α* and displaces IL-15/IL-15R*α* binding in a dose-dependent manner [21]. Therefore, we considered that detected IL-15R*α* is not forming complexes with endogenous IL-15. Measured levels of IL-15R*α* were significantly increased in RA compared with OA (a rheumatic nonautoimmune disease) suggesting a proinflammatory role in this disease. To determine the molecular weight of IL-15R*α* in synovial fluids, we captured it with a P8 peptide synthesized on TentaGel-S pearls. A band about 42 kDa was recognized in a western blot using an anti-IL-15R*α* antibody. The observed size corresponded to the previous report for soluble IL-15R*α* released from positive cells by a shedding process involving matrix metalloproteinases [9]. Presence of IL-15R*α* in the synovial fluids is a requisite to induce reverse signaling through membrane-bound IL-15. A mechanism which has been recently described in THP1 monocytic cells, PC-3 prostate carcinoma cells and in patients with head and neck cancer. There it is proposed that IL-15 anchored in plasma membranes acts as a receptor being capable to bind soluble IL-15R alpha then inducing MAPK and IL-6. MAPKs (ERK and p38) are a family of highly conserved serine/threonine kinases that have been described to play key regulatory roles in downstream signaling events leading to joint inflammation, and joint destruction including production of proinflammatory cytokines such as IL-6 [22]. Expression of IL-6 is increased in the synovium of patients with RA, and serum levels of IL-6 have been shown to correlate with clinical and laboratory markers of disease activity, and IL-6 has recently been validated as a target in RA [23]. Therefore, it is important to know if this signaling pathway induced by interaction between membrane IL-15 and IL-15R*α* takes place in RA to induce IL-6.

Although membrane IL-15 had been detected in synovial tissues from RA patients [18, 24], it has not been studied whether membrane IL-15 is bound to IL-15R*α* or exists as a membrane-anchored protein. In membrane IL-15 bound to IL-15R*α*, IL-15 is retained on the cell surface, and it is transpresented to IL-2R/15R*β*-*γ*c on nearby effector NK and T cells by the formation of an immunological synapse [25, 26]. Thus, IL-15/IL-15R*α* activates the JAK1/JAK3 and STAT3/STAT5 pathways to induce proliferation of T and NK cells, and this mechanism could limit exposure to circulating IL-15, that contributeS to the risk of autoimmunity [7]. In contrast, IL-15 R-independent membrane-bound IL-15 could act as a receptor inducing reverse signaling. In this current study, we found that after acid treatment most of IL-15 is present as a membrane-anchored protein, and a certain number of IL-15 molecules are bound to membrane IL-15 R*α* confirming the expression of membrane-anchored IL-15 on cells from synovial fluids.

Chronic joint inflammation is related to leukocytes infiltration in synovial compartment. The synovium of patients with established RA is expanded and contains large numbers of fibroblasts, macrophages, and highly differentiated T cells [27]. We observed at least three cell populations with different SSC/FCS characteristics by flow cytometry (Figure 3(a)). We could not perform double staining, but interestingly, IL-15 positive cells were present in the R1 region, and 95% of this population was CD3 positive and 83.8% were CD8 positive, suggesting, they were IL-15-positive T cells. This finding is in agreement of previous results by Miranda-Carús et al., who detected IL-15 on rheumatoid arthritis T cells [18].

To explore the production of the proinflammatory cytokine IL-6 in these patients, we quantified IL-6 levels in synovial fluids from RA and OA patients. Higher and significant concentrations of IL-6 were found in RA when compared with OA patients. This result is in agreement with a previous paper [28], but interestingly, we found a positive correlation (*r* = 0.61; *P* = 0.006) between high levels of IL-6 and high levels of IL-15R*α* in RA but not in OA. This data suggested that IL-15R*α* present in synovial fluids could be possibly inducing IL-6 through a reverse signaling pathway and then contributing to a proinflammatory medium in RA. To demonstrate that cells from synovial fluid could secrete IL-6 in response to IL-15R*α*, we performed an experiment in which cells from synovial fluid were incubated with or without IL-15R*α*. A significant increase of IL-6 was observed in the supernatant culture of cells treatment with IL-15R*α*.

This result reveals a possible proinflammatory role of soluble IL-15R*α* through reverse signaling. The presence of soluble IL-15 in synovial fluids from RA patients and its role in inducing migration of T cells and induction of TNF alpha is already known [8]. Possibly, both soluble and membrane IL-15 are implicated in the proinflammatory process through different pathways. Therefore, this finding might imply that different approaches would be necessary for an effective inhibition of IL-15 signaling in RA. Now, we will perform experiments to assess antagonist properties of the P8 peptide in this context.

In conclusion, we have detected soluble IL-15 alpha in synovial fluids, which is increased in RA in comparison to OA. In addition, it is positively correlated to IL-6 specifically in RA. These results suggested that IL-15R alpha could induce IL-6 in RA through its binding to membrane IL-15.

## Figures and Tables

**Table 1:** Patient demographics.

	RA (*n* = 18)	OA (*n* = 17)
Sex (M/F)	4/14	9/8
Age (years)	49 ± 14.19	64 ± 9.8
Disease duration (years)	13 ± 11	10 ± 2.6
Rheumatoid factor (±)	6/12	—
DAS28	4.37 ± 1.23	—
DMARD (MTX)	16	—

Demographics showing age, sex, and duration of disease, where available;

RF: rheumatoid factor status; DAS28: disease activity score; DMARD: disease-modifying antirheumatic drug.
